# Influences of claywater and greenwater on the skin microbiome of cultured larval sablefish (*Anoplopoma fimbria*)

**DOI:** 10.1186/s42523-020-00045-5

**Published:** 2020-08-04

**Authors:** Emily T. Dodd, Melissa L. Pierce, Jonathan S. F. Lee, Rachel S. Poretsky

**Affiliations:** 1 Department of Biological Sciences, University of Illinois at Chicago, 845 W. Taylor Street, Chicago, IL 60607 USA; 2 Environmental and Fisheries Sciences Division, Northwest Fisheries Science Center, National Marine Fisheries Service, National Oceanic and Atmospheric Administration, 7305 Beach Drive E, Port Orchard, WA 98366 USA

**Keywords:** Skin microbiome, Sablefish, Greenwater, Claywater, 16S rRNA gene, Aquaculture

## Abstract

**Background:**

The skin microbiome of marine fish is thought to come from bacteria in the surrounding water during the larval stages, although it is not clear how different water conditions affect the microbial communities in the water and, in turn, the composition and development of the larval skin microbiome. In aquaculture, water conditions are especially important; claywater and greenwater are often used in larval rearing tanks to increase water turbidity. Here, we explored the effects of these water additives on microbial communities in rearing water and on the skin of first-feeding sablefish larvae using 16S rRNA gene sequencing. We evaluated three treatments: greenwater, claywater, and greenwater with a switch to claywater after 1 week.

**Results:**

We observed additive-specific effects on rearing water microbial communities that coincided with the addition of larvae and rotifer feed to the tanks, such as an increase in *Vibrionaceae* in greenwater tanks. Additionally, microbial communities from experimental tank water, especially those in claywater, began to resemble larval skin microbiomes by the end of the experiment. The differential effects of the additives on larval sablefish skin microbiomes were largest during the first week, post-first feed. Bacteria associated with greenwater, including *Vibrionaceae* and *Pseudoalteromonas* spp., were found on larval skin a week after the switch to claywater. In addition to additive-specific effects, larval skin microbiomes also retained bacterial families likely acquired from their hatchery silos.

**Conclusions:**

Our results suggest that larval sablefish skin microbiomes are most sensitive to the surrounding seawater up to 1 week following the yolk-sac stage and that claywater substituted for greenwater after 1 week post-first feed does not significantly impact skin-associated microbial communities. However, the larval skin microbiome changes over time under all experimental conditions. Furthermore, our findings suggest a potential two-way interaction between microbial communities on the host and the surrounding environment. To our knowledge, this is one of the few studies to suggest that fish might influence the microbial community of the seawater.

## Background

The epidermal mucosal layer of marine fish is inhabited by a diverse assemblage of bacteria that aid in host health and survival [1, 2]. These microbes are the first line of defense against pathogens [1–5] and can reduce frictional drag during swimming [6–8]. During the early life stages, larval skin is colonized by microbes present in the water and on the chorion of the egg [1, 2, 9]. The skin microbiome diversifies over time to ultimately become distinct from the microbial communities in the surrounding seawater by adulthood [1, 10–14].

Skin microbiomes vary among marine fish species [13, 15]. Inter- and intra-individual, regional, and seasonal variation have been observed in some species [13, 14, 16, 17]. Extrinsic factors such as diet [18] and substances that are present in the water [19, 20] can also shift the skin microbiomes of some species of marine and freshwater fish, including larvae. These interactions remain unclear, however, and the effects of environmental conditions and seawater microbiota on the skin microbiome of marine fish species such as sablefish (*Anoplopoma fimbria*) are not well understood.

Sablefish are a marine cold-water species found in the deep waters along the continental slope in the northern Pacific Ocean [21–23]. Commonly referred to as black cod or butterfish, sablefish are highly valued in the seafood market and are considered a prime candidate for aquaculture. However, the larval stages of rearing pose a challenge due to high costs [24–27]. Water additives are commonly used in aquaculture to increase water turbidity, enabling larvae to better see their feed, navigate tanks more efficiently, and have higher rates of growth and survival than larvae reared in clear water [28–36]. Adding algae to seawater, creating a mixture known as “greenwater,” has benefits to larval feeding, behavior, growth, and survival [28, 29, 37–39]. However, algal paste is expensive, and growing algae can be labor intensive without consistent yield. It can also promote bacterial growth, including pathogens [40]. Clay has been identified as a potential substitute to algae. Claywater typically has a reduced abundance of *Vibrio* spp. [41, 42] and up to 90% cost reduction compared to greenwater, but research into its effects on larval growth and survival has yielded mixed results [25, 40–42].

For sablefish, greenwater in the first week of larval rearing, either alone or with low concentrations of clay, has led to greater growth and survival compared to only clay, and switching from greenwater to claywater during the second week produced 1.5 times greater larval growth compared to tanks receiving only greenwater [25]. These results suggest that benefits of greenwater in the first week of larval rearing may go beyond turbidity, perhaps helping to shape host-associated microbial communities. Because the fish skin microbiome plays a role in host health and survival and may be more sensitive to the conditions and bacteria in the surrounding seawater during the early life stages, it is important to understand how the fish skin microbiome develops and changes in response to water additives used during the larval stages.

Because algae and clay are known to affect the bacterial composition of water [40–42], we hypothesized that they would influence the bacteria on larval skin as well, particularly in the week of first-feeding when they may be more sensitive to environmental and diet changes [2, 9, 18]. Here, we investigated the interaction between microbial communities on the skin of first-feeding sablefish larvae and the surrounding tank water under three different additive treatments: claywater (CC), greenwater (GG), and greenwater with a switch to claywater after 1 week (GC). This work is part of a larger study; results on the effects of these treatments on the internal sablefish and tank biofilm microbiomes, as well as survival, are available in Pierce et al. [24]. Our findings have implications for larval skin microbiome development, interactions between host-associated microbial communities and the rearing water, and fish health and survival.

## Results

A total of 3,129,355 sequences and 17,336 OTUs were observed from 147 samples. Reads per sample ranged mostly from 2000 to 30,000, but three water samples from claywater tanks and one water sample from a greenwater tank had fewer than 1000 reads, with a claywater sample having the fewest number of reads (285). Algae, rotifer, and larval skin samples ranged from 20,038–39,092 reads per sample. Powdered clay and water samples ranged from 285 to 31,831 reads per sample, with none of the powdered clay samples having more than 700 reads.

### Larval sablefish skin microbiomes

**Fig. 1:**
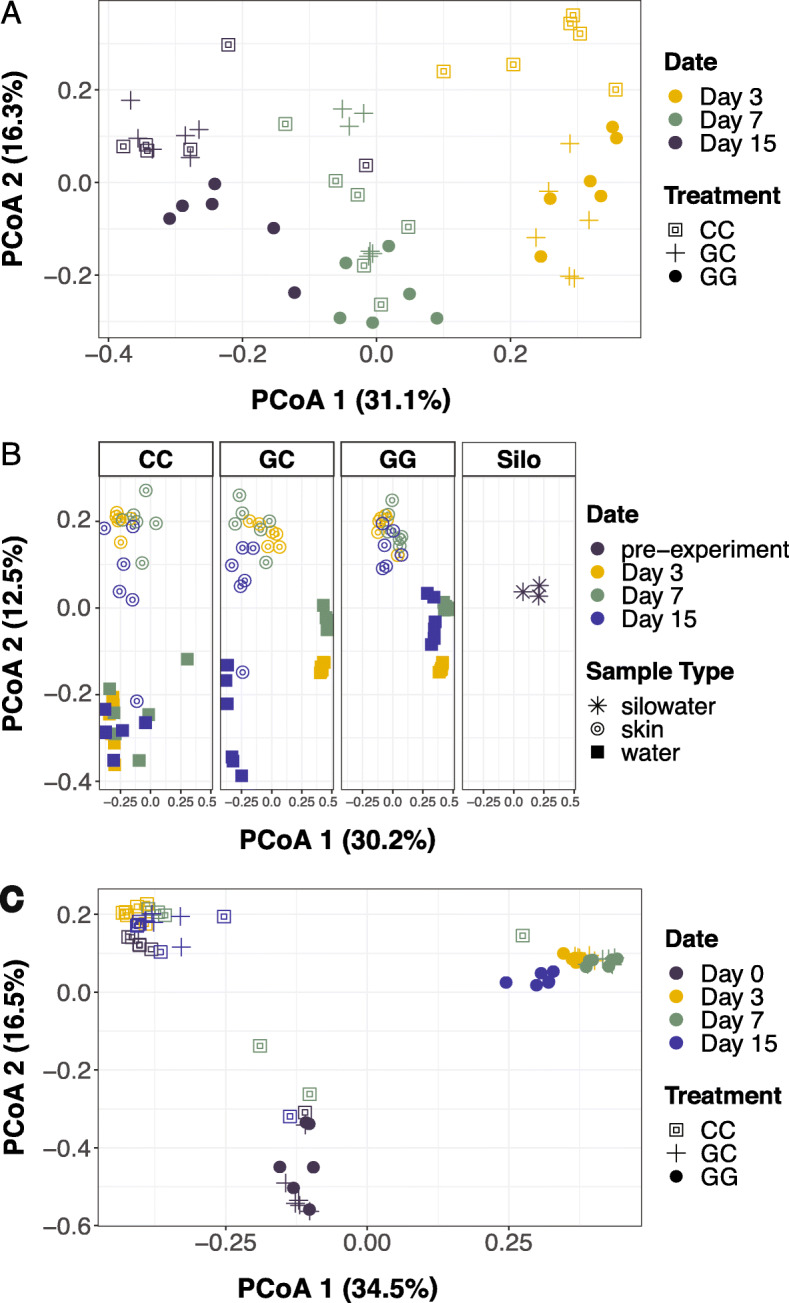
**a** Principle coordinates analysis (PCoA) ordination of Bray-Curtis distances between microbial communities on larval sablefish skin over time. **b** Bray-Curtis PCoA of microbial communities associated with larval skin, hatching silo water, and experimental tank water. **c** Bray-Curtis PCoA of tankwater microbial communities over time. Silo water was only sampled once prior to the experiment/stock-out (pre-experiment). Each point represents a single sample (*n* = 6 per treatment for each date and sample type. Pre-experiment silo water *n* = 3). CC = claywater for 15 days. GC = greenwater with a switch to claywater after 1 week. GG = greenwater for 15 days

**Fig. 2:**
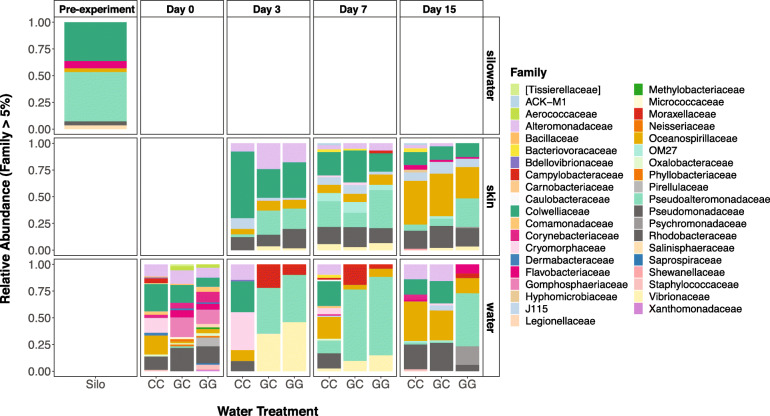
Relative abundances of bacterial families greater than 5% of the total abundance in larval fish skin, experimental tank water, and hatching silo water microbial communities, faceted by date and treatment (For each sample type and day, *n* = 6 per treatment. Pre-experiment silo water *n* = 3)

Microbial communities on larval sablefish skin changed significantly over time (PERMANOVA, *R*^*2*^ = 0.386, *p* < 0.001), with relatively low influence of water additive (*R*^*2*^ = 0.069, *p* = 0.002; Figs. 1a and 2) and no influence of parental cross (*R*^*2*^ = 0.028, *p* = 0.18; Additional file 1). The effects of the water additives on larval skin microbiome variation were largest during the first week post first-feed, especially on day 3 between CC and GG treatments (*R*^*2*^ = 0.431, *p* = 0.004; Additional file 1), although variation between treatments was low when phylogenetic distance was considered (*R*^*2*^ = 0.011, *p* = 0.99; Additional file 2).

Sablefish skin was more similar to water from the hatching silos than the experimental tank water (Fig. 1b). Specifically, many of the bacterial families that were abundant on larval skin were also abundant in the water of the hatching silos, including *Alteromonadaceae, Colwelliaceae*, *Oceanospirillaceae*, and *Pseudoalteromonadaceae* (Fig. 2). SourceTracker results indicated that this was especially true for greenwater treatments: the proportion of silo water microbial communities represented on larval skin samples on day 3 was an average of 0.11 in the CC treatment, 0.39 in the GC treatment, and 0.44 in the GG treatment. On day 15, the proportion of silo water communities represented on larval skin dropped below 0.1 in the CC and GC treatments, but remained at an average of 0.25 in the GG treatment (Additional file 3).

**Fig. 3:**
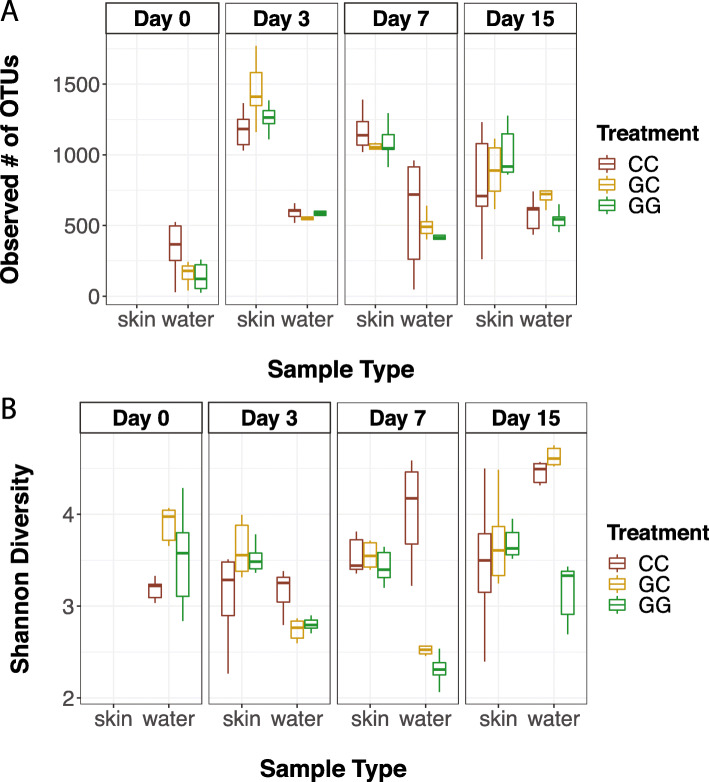
Effects of treatment and date on (**a**) the observed number of OTUs and (**b**) Shannon diversity of microbial communities on larval sablefish skin and in experimental tank water. Samples from larval skin were not taken on day 0. (For each date and sample type, *n* = 6 per treatment). CC = claywater for 15 days. GC = greenwater with a switch to claywater after 1 week. GG = greenwater for 15 days. Box plots represent median values with lower and upper hinges corresponding to the first and third quartiles

**Fig. 4:**
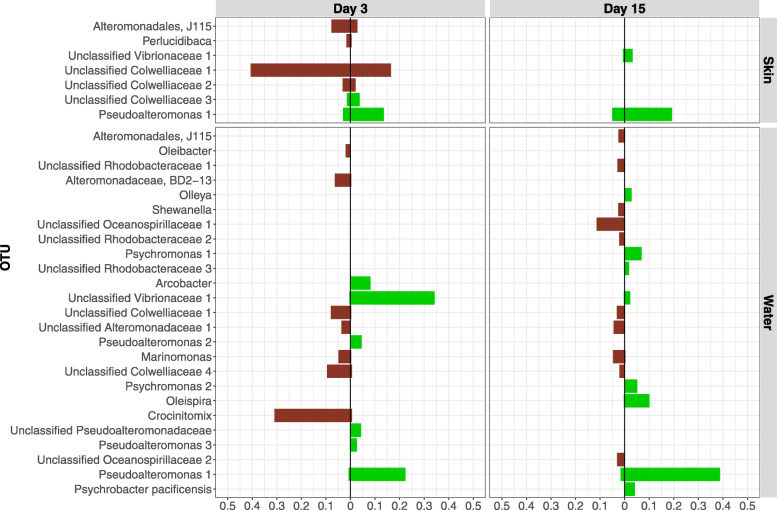
OTUs that are significantly different (*p* < 0.035) between claywater (CC) and greenwater (GG) treatments, faceted by sample type and date. The relative abundance of each OTU in the groups being compared is given on the x-axis. OTUs enriched in the CC treatment are represented by the brown color, and OTUs enriched in the GG treatment are represented by the green color. Different OTUs that have the same taxonomy are designated with different numbers (1–5), i.e., Unclassified Colwelliaceae 1 and Unclassified Colwelliaceae 2 are different OTUs. OTUs that share the same name and, if applicable, same number are the same OTU

Skin communities varied by treatment and time based on both microbial diversity and composition. On day 3, fish skin microbial communities from the GC treatment were significantly more diverse (lmer, β = 0.512, SE = 0.231, df = 45.00, t = 2.214, *p* = 0.032) and had significantly more OTUs (β = 275.17, SE = 109.50, df = 44.61, t = 2.51, *p* = 0.016) than fish skin communities in the CC treatment (Fig. 3a and b). Six OTUs were significantly different between skin communities from CC and GG treatments on day 3 compared to two OTUs on day 15. On day 3, two unclassified *Colwelliaceae* OTUs, a *J115* (*Alteromonadales*), and a *Perlucidibaca* were more abundant in skin communities from the CC treatment. A third unclassified *Colwelliaceae* and a *Pseudoalteromonas* were more abundant in skin communities from the GG treatment. The same *Pseudoalteromonas* OTU as well as an unclassified *Vibrionaceae* were more abundant in skin communities from the GG treatment than the CC treatment on day 15 (DESeq, *p* < 0.05; Fig. 4). PERMANOVA results also showed a significant difference between skin communities from GG and GC treatments on day 7 (Bray Curtis: *R*^*2*^ = 0.222, *p* = 0.027; Weighted UniFrac: *R*^*2*^ = 0.276, *p* = 0.008; Additional files 1 and 2), even though there were no OTUs that were significantly different between the two treatments on that day, suggesting that differences may be due to low abundance OTUs and/or there were differences in relative abundance rather than differences in specific organisms.

**Fig. 5:**
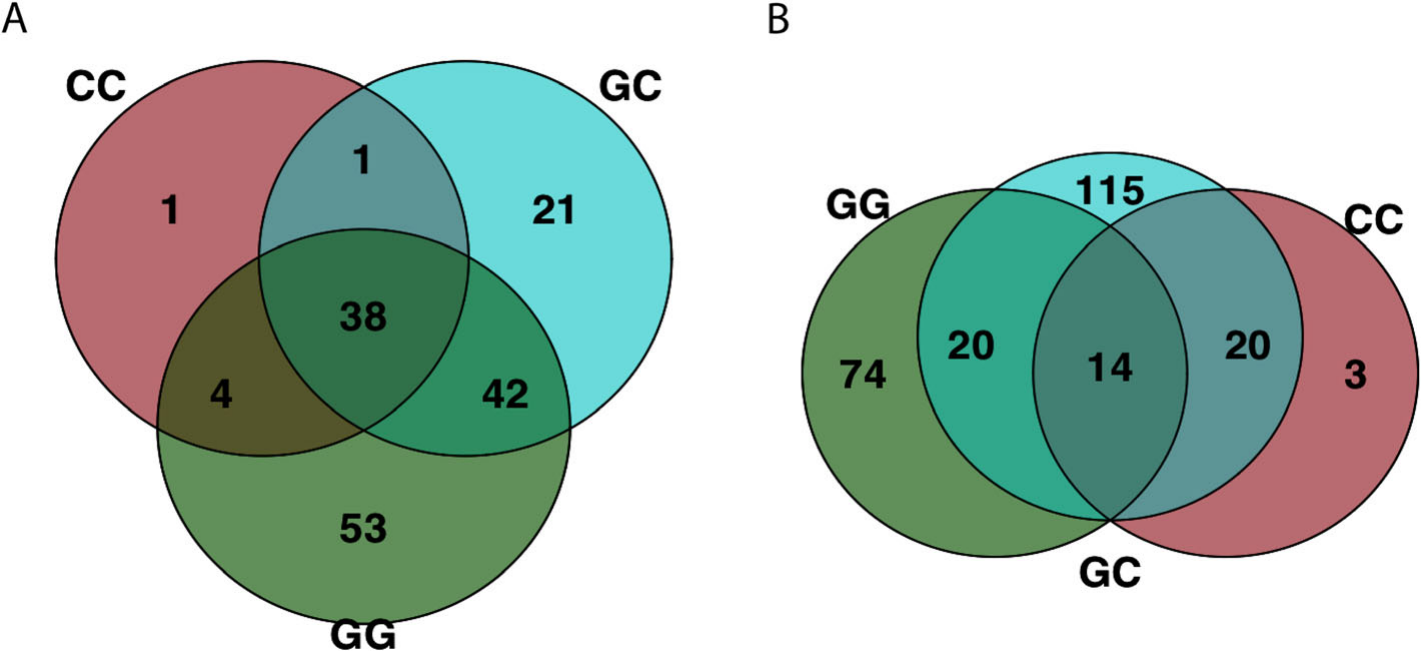
Core OTUs shared by 90% of samples from **a**) larval fish skin and **b**) tank water microbial communities in each treatment on day 15. On day 15, three true treatments were represented

OTUs associated with greenwater were found on larval skin a week after the switch to clay, including those classified as *Pseudoalteromonas*. There was no significant effect of treatment on larval skin microbiome variation on day 15 (PERMANOVA, *R*^*2*^ = 0.153, *p* = 0.22; Additional file 1), however, sablefish skin from the GC treatment shared more core OTUs with those from the GG treatment than the CC treatment (Fig. 5). The same *Vibrionaceae* OTU that was over six times more abundant in GG tank water than CC tank water throughout the experiment (DEseq, *p* < 0.001; Fig. 4) was also three times more abundant in larval skin from the GG treatment than the CC treatment on day 15 (log_2_ fold change = 2.296, SE = 0.809, *p* = 0.027; Fig. 4). Additionally, the same *Pseudoalteromonas* OTU was four times more abundant in GG microbial communities from both larval skin and tank water on day 3 and day 15, relative to the CC treatment (*p* < 0.05; Fig. 4).

Larval sablefish skin microbiomes were distinct from the surrounding water throughout the experiment (Fig. 1b, Additional file 1) and had at least 200 more OTUs than tank water of the same treatment (lmer; *p* < 0.001; Fig. 3a). This was less evident towards the end of the experiment, as sablefish skin and tank water microbiomes were more similar to each other on day 15 than on day 3, especially in claywater and when phylogenetic distance was considered (Fig. 1b, Additional files 1 and 2). While sablefish skin and tank water microbial communities were more similar on day 15 compared to day 3, they were still statistically distinct (PERMANOVA; *p* > 0.05; Additional files 1 and 2). The number of OTUs in larval sablefish skin communities decreased by at least 200 between day 3 and day 15 in all treatments (*p* < 0.05). In the GC treatment, the number of OTUs was reduced by at least 400 (β = − 569.67, SE = 105.83, df = 30.00, t = − 5.38, *p* < 0.001; Fig. 3a). On day 15, larval sablefish skin microbial communities across treatments shared 38 core OTUs (core OTU defined at 90% prevalence), and only 8 were also core OTUs from tank water communities. Core OTUs shared by only larval skin communities were unclassified members of *Cryomorphaceae*, *Halomonadaceae*, *J115* (order *Alteromonadales*), *OM27* (order *Myxococcales*), and *Vibrionaceae,* as well as *Arcobacter*, *Methylotenera mobilis*, *Perlucidibaca*, *ZD0117* (family *Alteromonadaceae*), *BD2–13* (family *Alteromonadaceae*), *Crocinitomix*, *Thalassomonas*, and *Phaeobacter*.

### Tank water microbial communities

Additives significantly influenced tank water microbial communities (PERMANOVA; *R*^*2*^ = 0.319, *p* < 0.001; Additional file 1) to a greater extent than they did larval skin. Additionally, changes over time were treatment specific. Between day 0 and day 3, there was a large shift in the microbial communities in tank water from all treatments, especially those with greenwater. At day 0, GC tank water communities were significantly more diverse than those in the CC treatment (lmer; β = 0.63, SE = 0.20, df = 57.25, t = 3.13, *p* < 0.001; Fig. 3b), but by day 3 there was a significant reduction in diversity in greenwater tanks (*p* < 0.05) and a significant increase in the number of OTUs in all tank water communities (*p* < 0.01; Fig. 3a and b) that remained throughout the experiment. On day 0, a *Devosia* OTU was enriched in the CC treatment, and a *Gomphosphaeriaceae* OTU was enriched in greenwater tanks (DESeq; *p* < 0.001; Additional file 4) compared to day 3.

On day 3 the most abundant families in CC tank water were *Alteromonadaceae*, *Colwelliaceae*, *Cryomorphaceae*, *Oceanospirillaceae*, and *Rhodobacteraceae*. The GG and GC tank water communities shifted to contain mostly *Campylobacteraceae*, *Pseudoalteromonadaceae*, and *Vibrionaceae* (Fig. 2). Most of the OTUs that were significantly different (DESeq; *p* < 0.05) between day 0 and day 3 were enriched on day 3, including *Crocinitomix*, *Oleibacter*, and *BD2–13* in the claywater tanks and *Pseudoalteromonas* and *Arcobacter* in the greenwater tanks (Additional file 4). Between GG and CC treatments, OTUs attributed to *Cronitomix, Oleibacter,* and *BD2–13* were significantly more abundant in CC tank water (*p* < 0.001), *Arcobacter* and *Phaeobacter* were significantly more abundant in GG tank water on day 3 (*p* < 0.001; Fig. 4). PERMANOVA results also showed a significant difference between GG and GC tank water communities on both day 3 (Bray Curtis: *R*^*2*^ = 0.463, *p* = 0.005; Weighted UniFrac: *R*^*2*^ = 0.588, *p* = 0.006) and day 7 (Bray Curtis: *R*^*2*^ = 0.324, *p* = 0.014; Weighted UniFrac: *R*^*2*^ = 0.495, *p* = 0.005; Additional files 1 and 2). There were four OTUs that were differentially abundant between GG and GC tank water communities during the first week of the experiment, including an enrichment of *Vibrionaceae*, *Pseudoalteromonas*, and *Oleispira* in GG tank water (DESeq; *p* < 0.05) and *Arcobacter* in GC tank water (*p* < 0.01; Additional file 5).

Tank water microbial communities across all treatments were more similar to fish skin communities on day 15 than they were on day 3 (Fig. 1b, Additional files 1 and 2). A *Shewanella* OTU was significantly enriched in claywater tanks, increasing from undetected on day 3 to a relative abundance of 2.64% on day 15 (*p* < 0.001). *Oleispira*, *Psychromonas, Psychrobacter pacificensis,* and *Olleya* OTU relative abundances were at least 10 times higher in GG tanks (*p* < 0.001; Fig. 4) on day 15 compared to day 3. In GC tank water communities, both the Shannon diversity index and number of observed OTUs increased by a factor of 1.5 by day 15 (lmer; *p* < 0.05; Fig. 3a, b), 8 days after the switch to claywater. Microbial communities in GC and CC tank water were not significantly different in terms of diversity on day 15, but GC tank water had significantly more OTUs than CC tank water (β = 209.33, SE = 96.75, df = 42, t = 2.16, *p* = 0.036; Fig. 3a). Tank water microbial communities across all treatments shared 14 core OTUs on day 15, which is less than half the number of core OTUs shared by larval skin communities (Fig. 5). Over half of the core OTUs in tank water communities were also core OTUs on larval skin, including members of *Pseudoalteromonas*, *Marinomonas*, and unclassified members of *Alteromonadaceae, Colwelliaceae,* and *Oceanospirillaceae*. Tank water core OTUs were members of *Olleya*, *Hyphomonadaceae*, and *Oleiphilaceae*.

## Discussion

Our study is novel in that we were able to observe longitudinal microbiome interactions between larval fish and water under different aquaculture treatment conditions. Our finding that microbial communities from tank water and larval skin become more similar to one another after 15 days highlights an interaction between larval skin and rearing water in the 2 weeks following larval stocking and first-feed. Because larval skin microbiomes retained many of the bacterial families present in the hatching silo water, and the temporal changes in the larval skin microbiomes were generally the same across treatments while the water microbiomes varied by treatment, it is possible the skin communities may have influenced those in the surrounding water. This adds a new perspective to what had previously been thought about water communities influencing fish skin [1, 2, 9]. Interactions between host-associated microbial communities and a host’s environment have been investigated for larval zebrafish, and point to the dynamic influence the host themselves may have in microbial dispersal [43, 44]. It is possible that the proliferation of *Vibrionaceae*, *Campylobacteraceae*, and *Pseudoalteromonadaceae* in greenwater tanks precluded skin-associated microbial communities from existing in a free-living state, but further studies are necessary to understand why claywater may be more accommodating for larval skin-associated communities.

Many of the genera found in tank water microbial communities, including anaerobic *Shewanella* spp., have been associated with both clay- and greenwater [45]. Our findings also agree with other studies that have showed more *Vibrionaceae* in greenwater tanks compared to claywater tanks [40–42]. Stuart et al. [41] observed an increase in *Vibrio* spp. in tanks supplemented with algal paste following addition of larvae and rotifer feed, so it is possible that the significant reduction in diversity in greenwater tank water and increase in observed OTUs in tank water from all treatments on day 3 may have been a result of the addition of larvae, live rotifer feed, and/or the accumulation of fecal matter/decaying material. It should be mentioned that the current study describes relative abundances, and shifts in vibrios and other bacterial taxa are not necessarily related to growth.

SourceTracker results indicate that rotifer feed and water additives were not significant contributors to the composition of larval skin communities. Their presence, however, still represents an input of organic matter to the system, which likely influenced the tank water communities. These data also implicate silo seawater as the largest source tested influencing the larval skin microbiome. Interestingly, community composition data for clay powder, algae paste, and rotifer feed from Pierce et al. [24] show that *Pseudoalteromonadaceae* and *Vibrionaceae*. are not present in high abundances (relative abundances < 5%), further supporting the idea that hatching silo seawater may impart a lasting influence on skin colonization. Further, experimental treatment (claywater vs. greenwater) impacted whether those silo-associated OTUs remained on the skin, indicating there may be a dynamic interplay with treatment that can’t be directly attributed to the community composition of algae paste or clay powder.

Because the dissimilarity between treatments and sample types was reduced when phylogenetic distance was considered, the variation we observed between treatments and sample types may be due to changes in closely related bacteria. This is consistent with observations from Chiarello et al. [13] who found that dissimilarity between the skin microbiome of three marine fish species vs. the surrounding water dropped by almost half when phylogenetic distance is considered. Even though there were many overlapping genera between microbial communities in rearing tank water and larval skin, skin-associated communities consistently remained distinct from the surrounding water. This is unsurprising because they are distinct habitats; however, it is likely that the communities between the two habitat types interact with each other. Research on zebrafish by Robinson et al. [46] showed migration of bacteria from the aquatic environment into the gut of germ-free larvae. As fish skin surfaces are in constant contact with the surrounding water, it is likely there are transmission interactions between their microbiomes as well. Further, research on Atlantic salmon in hatcheries has highlighted the link between tank and fish microbiomes, citing the skin microbiome as an intermediary [47].

The richness of the skin microbiome of sablefish larvae was significantly greater than that of the surrounding water. This has been reported previously in adult Atlantic salmon [18]. Many of the genera found on larval sablefish skin have been associated with the skin of other organisms including humans [48], aquatic frogs [49], humpback whales (*Megaptera novaeangliae*) [50], European eels (*Anguilla Anguilla*) [51], and other marine and freshwater fish [3, 6, 13, 14, 16, 52–54]. Additionally, genera found on larval sablefish skin in this study have also been found on egg surfaces [55], intestinal tracts [45, 56–58], and gills [56, 58] of first-feeding larvae, reared, and wild-caught adults of other marine fish species. This suggests there may be a skin-specific microbiome during the yolk-sac and first-feeding stages of larval rearing that is similar to microbiomes associated with fish hosts in similar habitats.

Our observation of *Pseudoalteromonas* as a dominant genus, as well as members of *Vibrionaceae*, on larval sablefish skin and hatching silo water is consistent with other studies that have identified members of these two genera with eggs and larvae of marine fish [55], including bacteria that may provide disease protection in unfed yolk-sac larvae [59]. Members of both *Pseudoalteromonas* and *Vibrionaceae* have been reported to produce antibiotic compounds effective against common marine pathogens [59–61], and Pham et al. [61] observed increased survival rates of *Litopenaeus stylirostris* shrimp larvae when *Pseudoalteromonas* strain NC201 is added to the rearing water. While higher survival in greenwater treatments cannot be directly attributed to the presence of these bacteria, these data provide a foundation for understanding how microbes may play a role in overall fish health in aquaculture. Pierce et al. [24], the first publication of this larger study, reports that larval survival in greenwater and greenwater switched to claywater treatments was significantly higher compared to claywater. The persistence of these bacterial genera on larval skin, even after switching to claywater, suggests that they may be important. The increased presence of *Pseudoalteromonas* and *Vibrionaceae* on larval skin from the greenwater treatments may be one of many reasons why first-feeding sablefish larvae had increased survival rates when reared in greenwater compared to claywater [24, 25]; however, further exploration of the *Pseudoalteromonas* and *Vibrionaceae* OTUs associated with greenwater in this study is necessary in order to assess their probiotic potential.

Our findings show that the substitution of greenwater with claywater after 1 week post first-feed has a minimal effect on the larval sablefish skin microbiome. This study suggests that the larval sablefish skin microbiome is most sensitive to extrinsic factors such as hatching and rearing conditions up to 1 week post first-feed and that tank water microbial communities may be influenced by host-associated communities. To better understand the influence of the surrounding environmental conditions on the developing larval sablefish skin microbiome, additional studies that include egg and yolk-sac stages are needed. Longer-term experiments would aid in confirming the effects of these water additives on larval skin and if they persist beyond 2 weeks post first-feed. Additionally, further research specifically targeting the influence of host-associated microorganisms on the surrounding water is necessary to better understand these potential two-way interactions.

## Conclusion

Microbial communities in tank water and larval sablefish skin both changed significantly in the 2 weeks following the addition of larvae and rotifer feed into the tanks, but tank water communities were more affected by the water additives than those on larval skin. Larval skin communities resembled those found on the skin of other hosts and were distinct from the experimental tank water, despite many overlapping genera. The largest treatment effects on larval skin were present in the first week post first-feed. OTUs associated with greenwater were found on larval skin a week after the switch to clay, including *Vibrionaceae* and *Pseudoalteromonas* spp., which may be one of many reasons why sablefish larvae have increased survival rates in greenwater. Microbial communities on larval skin were more similar to water from the hatching silos than experimental tank water, and experimental tank water began to reflect larval skin communities by the end of the experiment, suggesting a two-way interaction between microbes on larval skin and in the rearing water. Claywater and larval skin were dominated by *Alteromonadaceae, Colwelliaceae, Oceanospirillaceae, Pseudoalteromonadaceae*, and *Rhodobacteraceae*. Greenwater tanks had an increased relative abundance of *Vibrionaceae*. Tanks with greenwater had a decrease in diversity, and both clay- and greenwater tanks had significantly more OTUs following addition of larvae and rotifer feed. Our findings suggest that claywater replacing greenwater after the first week post first-feed does not disturb the larval sablefish skin microbiome and, interestingly, that skin-associated microbial communities may influence those in the water. Overall, our results contribute to the growing knowledge of the interaction between host-associated microbial communities and the surrounding environment in an aquaculture setting.

## Methods

### Experimental design

The experiment was conducted at the Manchester Research Station of the NOAA Northwest Fisheries Science Center. Broodstock and hatching details can be found in Cook et al. [26], and details on feeding, lighting, and tank set up can be found in Lee et al. [62] and Pierce et al. [24]. Fertilized eggs were kept in incubators, then moved to hatching silos 10 days post-fertilization, with a 60-s antibacterial treatment in 200 ppm Perosan™ (Zep Sales & Services, Kent, WA, USA) during transfer. Pre-feeding larvae (~ 46 days post-fertilization) were then stocked out into eighteen 37-L experimental tanks at a density of 300 larvae per tank and fed live rotifers. Each tank contained larvae from one of three parental crosses with six tanks per cross.

Three water additive treatments were tested over the course of 15 days: greenwater (GG), claywater (CC), and greenwater with a switch to clay after 1 week (GC). The treatments were distributed evenly across each parental cross, so that each parental cross had two replicates within each treatment with a total of six tanks per treatment.

Concentrated claywater was made by mixing Kentucky Ball Clay OM4 (Kentucky-Tennessee Clay Company, Roswell, GA, USA) with seawater using a commercial blender. Concentrated greenwater was made by hand-mixing *Nannochloropsis* Instant Algae (68 million cells mL^− 1^, Reed Mariculture, Campbell, CA, USA) and green dye (“Green Shade Color,” Esco Foods, San Francisco, CA, USA). The concentrated additives were aerated and delivered into the flow-through tanks via manifold by peristaltic pumps (Anko Products, Bradenton, FL, USA) that were activated by cycle timers (Cap Controllers, Perris, CA, USA) to achieve desired concentrations of greenwater (0.021 mL algae & 0.005 mL green dye L^− 1^ seawater) and claywater (12 mg L^− 1^ seawater). Seawater from Puget Sound flowed through each tank at a rate of 250 mL min^− 1^. Incoming seawater was UV-sterilized and filtered to 1 μm. Water temperature in the tanks was 12 °C at stocking and gradually increased to 15 °C over the course of 4 days. Water temperature remained 15 °C for the remainder of the experiment.

### Sampling

Water samples were taken from the hatching silos 3 days before larvae were stocked into the experimental tanks (pre-experiment). Experimental tank water was sampled 2 hours after larvae were stocked into the tanks (day 0), and both tank water and larval surfaces were sampled 3, 7, and 15 days post-stocking (days 3, 7, and 15). Samples on day 7 were taken before the switch to claywater in the GC treatment. Two 60-mL water samples per tank were taken at each time point and syringe-filtered onto Sterivex 0.22 μm filters (EMD Millipore Corp., Burlington, MA, USA). To sample larval skin and control for inter-individual variation, 8–12 larvae from each tank were collected and swabbed using sterile cotton tip swabs (Puritan Medical Products Co., Guilford, ME, USA) and stored in transport tubes. At the final time point, only the remaining survivors in the claywater (CC) treatment were sampled as three tanks only had one survivor, one tank had two survivors, and two tanks had over 10 survivors. Samples of the algal paste, clay powder, incoming seawater, and rotifer feed were also taken. All samples were stored at − 20 °C for further processing.

### DNA extraction, amplification, & sequencing

Bacterial DNA was extracted from water samples using the PowerWater Sterivex DNA Isolation Kit (Mo Bio Laboratories, Inc., Carlsbad, CA, USA) according to manufacturer protocol. Swab sample extractions were done using the PowerSoil -htp 96 Well Soil DNA Isolation Kit (Mo Bio Laboratories, Inc., Carlsbad, CA, USA) with an adapted protocol to increase DNA yield by reducing the volume of reagents used in the Inhibitor Removal Technology® (IRT) steps to 150 μL each, followed by 10 min incubation at 4 °C. DNA from the algal paste, clay powder, and rotifer samples was extracted using the PowerSoil DNA Isolation Kit (Mo Bio Laboratories, Inc., Carlsbad, CA, USA) according to manufacturer protocol. The V3-V4 hypervariable region of the 16S ribosomal ribonucleic acid (rRNA) gene was amplified using a target amplicon sequencing (TAS) protocol previously described by Green et al. [63] with alterations that include previously designed 341F/806R primers with CS1/CS2 linkers and GoTaq Hot Start Colorless Master Mix (Promega, Madison, WI, USA).

Amplicon libraries were prepared and sequenced using the Illumina MiSeq platform (V3 chemistry, 2 × 300 paired-end sequencing) at the DNA Services Facility at the University of Illinois at Chicago. The raw sequencing reads are available on the NCBI Sequence Read Archive (BioProject accession number: PRJNA431797). Forward and reverse reads were merged using PEAR (v. 0.9.10), quality-filtered, and processed using the Quantitative Insights Into Microbial Ecology (QIIME, v. 1.9.1) pipeline [64]. Chimeric sequences were identified using UCLUST and removed. De novo operational taxonomic unit (OTU) assignment was done at 97% sequence similarity using the UCLUST method. The most abundant sequence of each OTU was used to generate a representative set of OTU sequences, and taxonomy was assigned to the representative set using Greengenes (13_8 release) [65]. Singletons, doubletons, and sequences identified as chloroplasts and mitochondria were removed prior to analysis. To avoid losing data that would be useful in comparing greenwater and claywater, samples were not rarefied [66].

### Statistical analysis

Statistical analyses were carried out using R statistical software (v. 3.4.4) with phyloseq (v. 1.22.3) [67], ape (v. 5.1) [68], vegan (v. 2.4–4), lme4 (v. 1.1–14) [69, 70], and DESeq2 (v. 1.18.1) packages [71, 72]. Core OTUs found in 90% of the samples were identified using the microbiome package (v. 1.0.0) [73], and Venn Diagrams of shared OTUs were generated using the VennDiagram package (v. 1.6.18) [74]. Principle coordinates analysis (PCoA) was used on Bray-Curtis [75] and Weighted UniFrac [76] distance matrices; both consider OTU identity and relative abundance, but UniFrac takes into consideration phylogenetic relationships. To test for the effect of sample type, water treatment, and time (date), permutational multivariate analyses of variance (PERMANOVAs) were run on each distance matrix using the adonis function [77]; larval parental cross was also included as a factor in PERMANOVAs on distance matrices containing only samples from larval skin. Homogeneity of variances were estimated with betadisper, which uses a multivariate analogue of Levene’s test, and tested with permutest, an ANOVA-like test with the null hypothesis that there is no difference in dispersion between groups. The function TukeyHSD was used to create confidence intervals around the differences between the mean dispersion estimates using Tukey’s ‘Honest Significant Difference’ method. Results from PERMANOVA models that did not have variance homogeneity were still reported, as they had a balanced design and are still considered admissible [78].

Diversity and richness (observed number of OTUs) were estimated using the estimate_richness function in the phyloseq package. The effects of sample type, treatment, and time on richness and Shannon index values were modeled with a linear mixed effects model with a random effect of tank. *P*-values from those models were obtained using the lmerTest package (v. 3.0–1) [79]. Differential abundance analysis of OTUs with DESeq2 was done using R code from the phyloseq tutorial, “Differential Abundance for Microbiome Data” [66, 67, 71]. This method uses size factors estimated from the geometric means and dispersion estimates to test for significance of coefficients in a negative binomial generalized linear model (GLM), which takes into consideration the excess zeroes and overdispersion often seen in microbial count data. Differential abundance analysis was carried out only on OTUs that have an abundance greater than 1% of the total abundance of all OTUs present in the samples being compared. The *p*-values presented from this test were adjusted for multiple-inference using the Benjamini-Hochberg correction within the DESeq function. The SourceTracker2 package, using theory originally described in Knights et al. [80], was used to estimate the proportion of microbial communities associated with rotifer feed, clay powder, algal paste, incoming seawater, tank water, and hatching silo water that are represented in larval skin communities.

## Supplementary information

**Additional file 1 **PERMANOVA results of the Bray-Curtis distances of larval fish skin and tank water microbial communities. Homogeneity of variances was estimated and tested, with significant *p*-values listed in parentheses. The effects of date and treatment were tested on subsets of the data containing only samples from fish skin or tank water. The effect of parental cross was tested on a subset of the data containing samples from fish skin. Each test was conducted using 1000 permutations. To maintain a balanced design, water samples taken at stock-out (Day 0) were not included in models comparing tank water and fish skin. In the first column, italics indicates the samples included in the model, and bold indicates the factors that are being tested. If the model contains only fish skin (*S*) or water samples (*W*), it is indicated in italics in the second column.

**Additional file 2 **PERMANOVA results using adonis function in R statistical software on the Weighted UniFrac distances of larval fish skin and tank water microbial communities that were calculated with UniFrac. Homogeneity of variances was estimated using betadisper and tested using permutest with significant p-values listed in parentheses. The effects of date and treatment were tested on subsets of the data containing samples from only fish skin or tank water. The effect of parental cross was tested on a subset of the data containing only samples from fish skin. Each test was conducted using 1000 permutations. To maintain a balanced design, water samples taken at stock-out (Day 0) were not included in models comparing tank water and fish skin. In the first column, italics indicates the samples included in the model, and bold indicates the factors that are being tested. If the model contains only fish skin (*S*) or tank water (*W*) samples, it is indicated in italics in the second column.

**Additional file 3.** SourceTracker estimates of proportions of microbial communities associated with hatching silo water, experimental tank water, algal paste, powdered clay, incoming seawater, and rotifer feed that are represented on larval sablefish skin on day 3 and day 15, averaged across all replicates. Analysis was done with burn-in of 1000 and no rarefication.

**Additional file 4 **DESeq results and taxonomy of OTUs that are significantly different (*p* < 0.05) in experimental tank water microbial communities between day 0 and day 3, ordered by log2 fold change for each treatment. *P*-values are adjusted using the default Benjamini-Hochberg correction within the DESeq function. OTUs with positive log2 fold changes are enriched on day 0, and OTUs with negative log2 fold changes are enriched on day 3.

**Additional file 5 **DESeq results and taxonomy of OTUs that are significantly different (*p* < 0.05) in tankwater microbial communities between the GG and GC treatments on day 3 and day 7, ordered by log2 fold change for each day. *P*-values are adjusted using the default Benjamini-Hochberg correction within the DESeq function. OTUs with positive log2 fold changes are enriched in the GG treatment, and OTUs with negative log2 fold changes are enriched in the GC treatment.

## Data Availability

The raw sequences from this study are available on the NCBI Sequence Read Archive (BioProject accession number: PRJNA431797).

## Abbreviations

16S rRNA: 16S ribosomal ribonucleic acid
ANOVA: Analysis of Variance
CC: Claywater
DESeq: Differential Expression Analysis for Sequence Count Data
df: Degrees of freedom
GC: Greenwater switched to claywater after one week
GG: Greenwater
GLM: Generalized Linear Model
IACUC: Institutional Animal Care and Use Committee
lmer: Linear Mixed-Effects Model
NCBI: National Center for Biotechnology Information
NOAA: National Oceanographic and Atmospheric Administration
OTU: Operational Taxonomic Unit
PEAR: Paired-End Read Merger
PERMANOVA: Permutational Multivariate Analysis of Variance
PCoA: Principle Coordinates Analysis
QIIME: Quantitative Insights Into Microbial Ecology
SE: Standard error
