# Supplementary material for: Influences of claywater and greenwater on the skin microbiome of cultured larval sablefish (Anoplopoma fimbria)
Source: Anim Microbiome. 2020 Aug 4;2:27. doi: 10.1186/s42523-020-00045-5 (PMC7807797; doi:10.1186/s42523-020-00045-5)
Supplement: Supplementary file 1 — Additional file 1 PERMANOVA results of the Bray-Curtis distances of larval fish skin and tank water microbial communities. Homogeneity of variances was estimated and tested, with significant p-values listed in parentheses. The effects of date and treatment were tested on subsets of the data containing only samples from fish skin or tank water. The effect of parental cross was tested on a subset of the data containing samples from fish skin. Each test was conducted using 1000 permutations. To maintain a balanced design, water samples taken at stock-out (Day 0) were not included in models comparing tank water and fish skin. In the first column, italics indicates the samples included in the model, and bold indicates the factors that are being tested. If the model contains only fish skin (S) or water samples (W), it is indicated in italics in the second column. [file 42523_2020_45_MOESM1_ESM.pdf]

	R^2	df	**Pseudo *F* ratio**	**P-Value**	**Homogeneity of variances**
**Date**	*S*: 0.386	2	18.214	< 0.001 *	Yes
	W: 0.220	2	19.808	< 0.001 *	Yes
with Day 0 samples	*W:* 0.276	3	18.143	< 0.001 *	Yes
CC & GG	*S:* 0.403	2	12.848	< 0.001 *	Yes
	W: 0.168	2	8.569	< 0.001 *	Yes
CC only	*S:* 0.429	2	5.633	< 0.001 *	Yes
	W: 0.370	2	4.413	< 0.001 *	No (0.030)
GC only	*S:* 0.532	2	8.515	< 0.001 *	Yes
	W: 0.834	2	37.781	< 0.001 *	No (< 0.001)
GG only	*S:* 0.572	2	10.014	< 0.001 *	Yes
	W: 0.774	2	25.666	< 0.001 *	No (0.014)
**Treatment**	*S*: 0.069	2	3.232	< 0.002 *	Yes
	W: 0.319	2	28.789	< 0.001 *	Yes
with Day 0 samples	*W:* 0.205	2	20.208	< 0.001 *	Yes

CC & GG	*S:* 0.084	1	5.335	< 0.001 *	No (0.014)
	W: 0.403	1	41.077	< 0.001 *	Yes
Day 0	*W:* 0.372	2	4.448	< 0.01 *	Yes
Day 0, CC & GG	*W:* 0.349	1	5.370	< 0.01 *	Yes
Day 0, GC & GG	*W:* 0.066	1	0.710	> 0.5	Yes
Day 3	*S:* 0.464	2	6.504	< 0.001 *	Yes
	W: 0.930	2	99.803	< 0.001 *	No (0.009)
Day 3, CC & GG	*S:* 0.431	1	7.562	< 0.01 *	Yes
	W: 0.930	1	133.370	< 0.01 *	No (0.008)
Day 3, GC & GG	*S:* 0.146	1	1.706	> 0.1	Yes
	W: 0.463	1	8.616	< 0.01 *	Yes
Day 7	*S:* 0.166	2	1.496	> 0.1	Yes
	W: 0.530	2	8.448	< 0.001 *	No (< 0.001)
Day 7, CC & GG	*S:* 0.123	1	1.400	> 0.15	Yes

	W: 0.463	1	8.613	< 0.01 *	No (< 0.001)
Day 7, GC & GG	*S:* 0.222	1	2.856	< 0.03 *	Yes
	W: 0.324	1	4.786	< 0.03 *	Yes
Day 15	*S:* 0.153	2	1.355	> 0.1	Yes
	W: 0.605	2	11.499	< 0.001 *	Yes
Day 15, CC & GG	*S:* 0.141	1	1.637	> 0.05	Yes
	W: 0.583	1	14.008	< 0.01 *	Yes
Day 15, CC & GC	*S:* 0.050	1	0.527	> 0.9	Yes
	W: 0.103	1	1.150	> 0.2	Yes
**Date x Treatment**	*S*: 0.081	4	1.908	< 0.01 *	Yes
	W: 0.211	4	9.524	< 0.001 *	No (< 0.001)
with Day 0 samples	*W:* 0.214	6	7.021	< 0.001 *	No (< 0.001)
CC & GG	*S:* 0.043	2	1.358	> 0.1	Yes
	W: 0.134	2	6.824	< 0.001 *	No (0.002)

**Sample Type**	0.141	1	44.211	< 0.001 *	No (< 0.001)
CC	0.197	1	12.228	< 0.001 *	Yes
CC, Day 3	0.729	1	26.904	< 0.01 *	No (0.021)
CC, Day 15	0.247	1	3.273	< 0.01 *	Yes
GC	0.206	1	26.338	< 0.001 *	Yes
GC, Day 3	0.801	1	40.302	< 0.01 *	No (< 0.001)
GC, Day 15	0.398	1	6.607	< 0.01 *	Yes
GG	0.326	1	43.526	< 0.001 *	Yes
GG, Day 3	0.855	1	59.093	< 0.01 *	No (< 0.001)
GG, Day 15	0.539	1	11.713	< 0.01 *	Yes
**Sample Type x Date**	0.077	2	12.126	< 0.001 *	Yes
CC	0.099	2	3.062	< 0.01 *	Yes
GC	0.216	2	13.821	< 0.001 *	No (< 0.001)
GG	0.151	2	10.092	< 0.001 *	No (0.002)
**Parental Cross**	*S*: 0.028	2	1.321	> 0.1	Yes
