# Supplementary material for: Influences of claywater and greenwater on the skin microbiome of cultured larval sablefish (Anoplopoma fimbria)
Source: Anim Microbiome. 2020 Aug 4;2:27. doi: 10.1186/s42523-020-00045-5 (PMC7807797; doi:10.1186/s42523-020-00045-5)
Supplement: Supplementary file 3 — Additional file 3. SourceTracker estimates of proportions of microbial communities associated with hatching silo water, experimental tank water, algal paste, powdered clay, incoming seawater, and rotifer feed that are represented on larval sablefish skin on day 3 and day 15, averaged across all replicates. Analysis was done with burn-in of 1000 and no rarefication. [file 42523_2020_45_MOESM3_ESM.pdf]

Source	**CC**		**GC**		**GG**	
	Day 3	*Day 15*	*Day 3*	*Day 15*	*Day 3*	*Day 15*
**silo water**	0.11	0.05	0.39	0.05	0.44	0.25
**tank water**	0.66	0.65	0.26	0.86	0.34	0.30
**algae**			0.00	0.02	0.00	0.01
**clay**	0.16	0.00		0.00		
**seawater**	0.00	0.03	0.00	0.01	0.00	0.02
**rotifer**	0.00	0.16	0.06	0.00	0.06	0.02
**unknown**	0.07	0.10	0.28	0.06	0.16	0.41
